# Solar-Powered Iron Electrocoagulation for Paracetamol Removal: Optimization, Colloidal Evidence, and Techno-Economic Assessment

**DOI:** 10.1021/acsomega.6c05565

**Published:** 2026-09-26

**Authors:** Ángel José De la Cruz Falcón, Rosangela Bergamasco, Joseane Debora Peruço Theodoro, Anieval Cirilo Peña Rojas, Magaly De La Cruz Rios, Artur Veloso Domingos, Cristiano António Colher

**Affiliations:** † Department of Chemical Engineering, 42487 State University of Maringá, Avenida Colombo 5790, Maringá, Paraná 87020-900, Brazil; ‡ Department of Chemical Engineering, Federal University of Technology − Paraná (UTFPR), Avenida dos Pioneiros 3131, Londrina, Paraná 86036-370, Brazil; § Faculty of Engineering, Universidad Nacional Del Centro Del Perú, Avenida Mariscal Castilla 3909, El Tambo, Huancayo 12006, Peru

## Abstract

The occurrence of paracetamol in water motivates the development of treatment processes that combine contaminant removal with reduced dependence on grid electricity. Iron-electrode electrocoagulation was optimized using a regulated direct-current power supply, a face-centered central composite design, and response surface methodology. The initial paracetamol concentration (0.1–0.3 mg·mL^–1^), reaction time (10–30 min), current density (0.95–6.67 A·m^–2^), and initial pH (3.0–10.0) were evaluated. The quadratic model was statistically significant (F = 3.072; p = 0.0293; R^2^ = 0.7818; adjusted R^2^ = 0.5273) and revealed significant interactions between current density and pH, concentration and current density, concentration and pH, and reaction time and pH. The highest experimental removal was 90.7%, obtained at 0.2 mg·mL^–1^, 30 min, 3.81 A·m^–2^, and pH 6.5, with a residual concentration of 18.6 mg·L^–1^. Near-zero ζ-potential values and floc growth up to approximately 44 μm were consistent with charge neutralization and sweep coagulation, although they did not establish a unique removal mechanism. The selected condition was subsequently operated using a configuration comprising a photovoltaic module, an MPPT controller, and a battery; photovoltaic supply was not a factor in the experimental design. At the maximum-removal condition, both configurations exhibited the same reactor energy consumption (0.018315 kWh·m^–3^) and theoretical iron dissolution (0.001737 kg·m^–3^). However, when a marginal purchase price of zero was assigned to photovoltaic electricity, the solar configuration was more cost-efficient than the conventional configuration: the partial variable cost decreased from BRL 0.013482·m^–3^to BRL 0.000495·m^–3^, corresponding to a 96.33% reduction. This comparison excludes photovoltaic system capital investment, battery replacement, maintenance, sludge management, labor, and life-cycle costs. The results identify a favorable operating region at the batch scale, although the resulting residual concentration does not demonstrate compliance with a discharge criterion.

## Graphical abstract

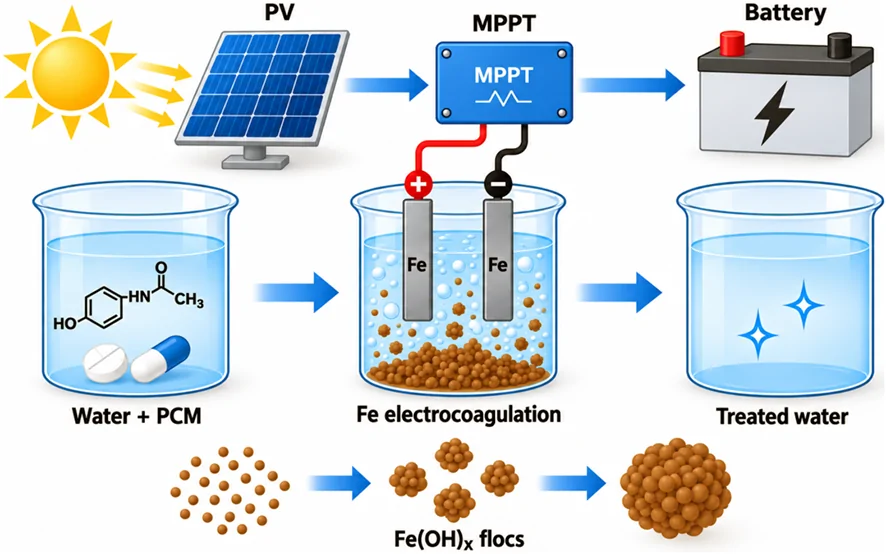

## 1 Introduction

Pharmaceutical compounds have been reported in surface water, groundwater, and industrial and municipal effluents.^1–3^ Their environmental persistence depends on molecular properties, use patterns, transformation, and the performance of treatment plants.^1,2^ Consequently, occurrence and treatment claims should be linked to compound-specific evidence rather than generalized across all pharmaceuticals.^2,3^

Paracetamol, also known as acetaminophen, is one of the pharmaceuticals most frequently detected in aquatic environments, as it is among the most widely consumed analgesic and antipyretic drugs worldwide. Its extensive use, together with its incomplete removal in conventional treatment systems, promotes its recurrent occurrence in hospital and municipal effluents. The persistence of this compound in treated waters and receiving bodies highlights the limitations of conventional biological processes and poses a relevant challenge for the development of more efficient treatment technologies from a chemical engineering perspective.^4–6^

Paracetamol removal by conventional biological treatment is strongly dependent on the matrix and plant configuration. Reported values include more than 80% removal during anaerobic sludge digestion and 97.1–98% removal in activated-sludge or full-scale sewage treatment; nevertheless, measurable residual concentrations may remain when influent loads are high.^7,8^ These data show why percentage removal must be interpreted together with influent and residual concentrations and why a complementary treatment step may still be relevant for concentrated pharmaceutical wastewater.

In this context, electrochemical processes have been applied to pharmaceutical-contaminant treatment, with electrocoagulation (EC) offering in situ coagulant generation, limited external coagulant addition, and compatibility with direct-current renewable electricity.^9,10^ In electrocoagulation using iron electrodes, anodic dissolution generates Fe^2+^ and Fe^3+^ species which, upon hydrolysis, form metal hydroxides and oxyhydroxides capable of inducing colloidal destabilization, adsorption, and sweep coagulation. These mechanisms promote the capture and subsequent removal of organic contaminants, as documented in studies on the fundamentals and applications of the process.^11,12^

However, the performance of electrocoagulation critically depends on operational variables such as reaction time, current density, initial pH, and mixing intensity. These parameters govern the generation of coagulant species, iron speciation, floc formation and growth, and the overall efficiency of the process. Given that the interaction between these factors exhibits nonlinear behavior, the application of robust statistical methodologies is required to simultaneously optimize multiple parameters and minimize energy consumption.^12–16^

Photovoltaic (PV) electricity can reduce dependence on the grid in electrocoagulation applications, particularly where solar irradiation is high or grid access is limited.^10,17,18^ However, the PV system does not alter the electrocoagulation mechanism: the reactor operating variables should first be optimized to define the required electrical dose, after which the PV module, controller, and storage capacity can be sized for that demand. Other sustainability strategies include minimizing electrical dose, extending electrode life, and using characterized scrap metal as sacrificial electrodes; the latter may reduce virgin-material demand but introduces concerns about composition, passivation, contaminant release, and reproducibility.^19,20^

Single-variable optimization approaches are insufficient to capture the coupled nature of these electrochemical and colloidal phenomena. In this regard, Response Surface Methodology (RSM) has emerged as an effective statistical tool for modeling and optimizing complex multivariable processes. This methodology enables the simultaneous evaluation of linear, quadratic, and interaction effects with a reduced number of experiments, facilitating the identification of optimal operating conditions and improving overall system performance.^21–23^ However, studies that systematically integrate statistical optimization of solar electrocoagulation with mechanistic analysis based on colloidal stability indicators and comparative techno-economic evaluation remain limited.

The motivation for this study was to determine whether iron electrocoagulation could remove paracetamol while defining an electrical demand compatible with a battery-buffered photovoltaic supply. The objectives were to (i) quantify the effects and interactions of initial concentration, reaction time, current density, and pH using a Face-Centered Central Composite Design (FCCCD) and RSM; (ii) relate removal to ζ-potential and particle-size evolution; and (iii) compare the reported partial variable costs of conventional and PV operating scenarios while explicitly stating the cost boundary. The contribution lies in integrating interaction-based process optimization, colloidal evidence, and a bounded power-supply comparison for paracetamol treatment; no claim that the PV source itself improves removal is made.

## 2 Methodology

### 2.1 Reagents and Solution Preparation

Paracetamol (acetaminophen, ≥99%, analytical grade) was used as the model pharmaceutical contaminant without further purification. Synthetic solutions were prepared in ultrapure water (18.2 MΩ·cm, Milli-Q system) at 25 ± 2 °C, within the concentration range defined by the experimental design (0.1–0.3 mg·mL^–1^).

NaCl (≥99.5%, analytical grade) was used as the supporting electrolyte to ensure adequate conductivity of the medium, and its concentration was kept constant throughout all experiments. The initial pH (3–10) was adjusted immediately before each experiment using diluted HCl or NaOH solutions.

Table 1 presents the most relevant physicochemical properties of paracetamol used as the model contaminant, including its molecular formula, molecular weight, purity, water solubility, melting point, p*K*_a_, and zeta potential in water. These parameters provide a basis for interpreting its stability in solution and its response to electrochemical treatment.

**Table 1:** Physicochemical Properties of Paracetamol Used in the Present Study

Parameter	Value	Unit
Molecular formula	C_8_H_9_NO_2_	–
Molecular weight	151.16	g·mol^–1^
Purity	≥99	%
Water solubility (25 °C)	14	g·L^–1^
Melting point	169–170	°C
p*K*_a_ (25 °C)	9.5	–
Zeta potential in water (25 °C)*	–20 ± 3	mV

### 2.2 Analytical Methods

Paracetamol concentration was determined by UV–Vis spectrophotometry (Hach DR 5000, USA) at a wavelength of λ = 243 nm, using a quartz cuvette with a 1 cm optical path length. The calibration curve was constructed within the concentration range of the prepared solutions, yielding the following linear regression model:

1 A=aC+b

where A is the absorbance and C is the paracetamol concentration (mg·mL^–1^). The coefficient of determination (R^2^) was higher than 0.99, indicating good linearity of the method.

The removal efficiency (R,%) was calculated as follows:

2 $$R\left(\%\right)=\left(\frac{Ci-Cf}{Ci}\right)x100$$

where C_i_ is the nominal initial concentration established during solution preparation and C*f* is the final concentration determined by UV–Vis spectrophotometry (mg·mL^–1^). Initial absorbance was not used to replace the gravimetrically prepared C_i_ in the removal calculation; this analytical limitation is considered when interpreting the reported percentages.

The zeta potential (ζ, mV) and hydrodynamic diameter (nm) were measured using a Malvern Zetasizer Nano ZS (Malvern Instruments, UK), equipped with a 4 mW He–Ne laser (λ = 633 nm, scattering angle = 173°). Measurements were conducted at 25 ± 2 °C. Prior to analysis, samples were filtered through 0.45 μm membranes when necessary to remove large particles not representative of the colloidal system.

The current (A) and voltage (V) were measured at the reactor terminals using a digital multimeter (Minipa ET-2042C, Brazil). Under PV operation, the battery buffered short-term irradiance variability, but no active constant-current controller was used; therefore, current was monitored rather than imposed.

### 2.3 Electrocoagulation System

Electrocoagulation (EC) experiments were conducted in a batch-mode glass reactor with an effective working volume of 2.0 L. Four iron plates (purity ≥99.5%) were used as sacrificial electrodes (anode and cathode), each with dimensions of 7.5 × 4.0 × 0.3 cm. The interelectrode spacing was fixed at 2 cm, according to the experimental conditions established in the statistical design (Figure 1).

**Figure 1:**
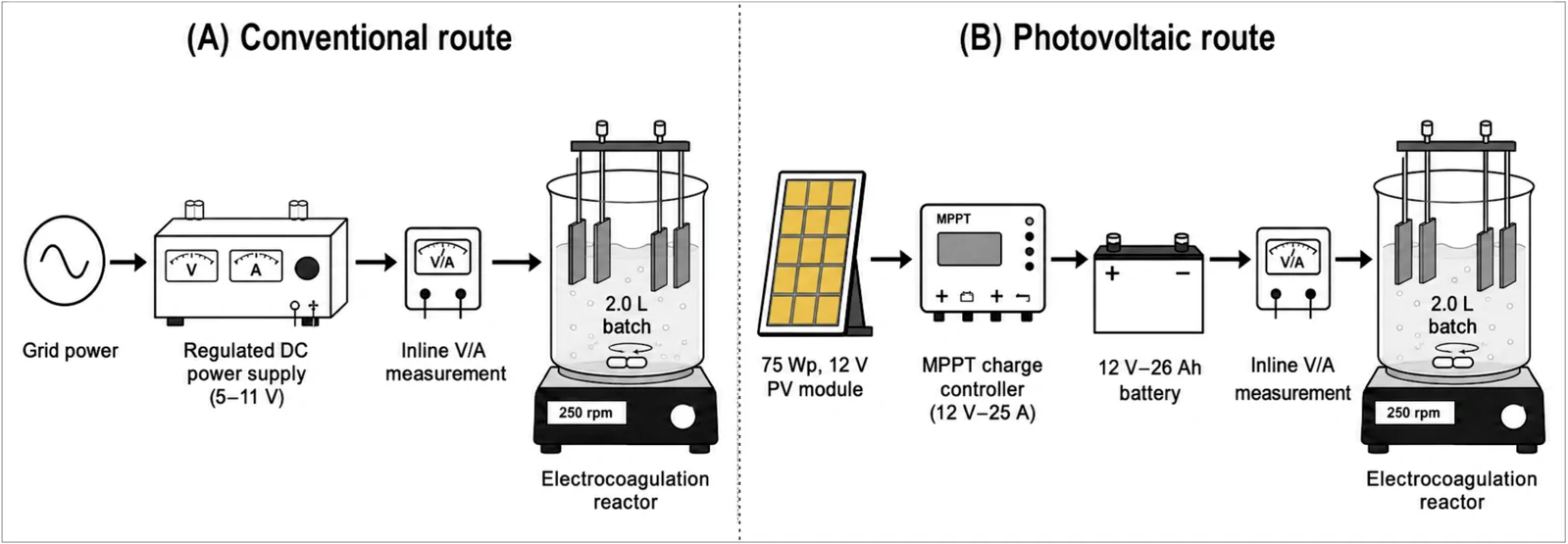
Separate electrical configurations evaluated. (A) Conventional route: grid-powered regulated DC supply → inline voltage/current measurement → electrocoagulation reactor. (B) Photovoltaic route: 75 Wp PV module → MPPT charge controller → 12 V battery → inline voltage/current measurement → reactor. The laboratory DC power supply was not included in the PV route; current was monitored but not actively maintained at a constant value.

Direct current (DC) was supplied through two separate configurations: (i) a conventional regulated laboratory DC power supply operating at 5–11 V and connected directly to the reactor; and (ii) a photovoltaic (PV) route consisting of a 75 Wp, 12 V module (Sova Solar SS70P), a 12 V–25 A maximum power point tracking (MPPT) charge controller, and a 12 V–26 Ah sealed maintenance-free battery. In the PV route, the module charged the battery through the MPPT controller and the battery supplied the reactor; the laboratory DC power supply was not part of this electrical path.

Continuous mixing was maintained using a magnetic stirrer to ensure adequate dispersion of electro-generated coagulant species, thereby promoting colloidal destabilization, aggregation, and floc formation during the treatment process.

Regardless of the energy source, the fundamental electrochemical behavior of the system remains unchanged. The process is initiated by the anodic oxidation of iron, releasing ferrous species into the aqueous medium:

3 $$Anode:\;Fe\left(s\right)\to Fe^{+2}\left(aq\right)+2e^{-}$$

Ferrous species may subsequently oxidize to Fe^3+^ and undergo hydrolysis, forming metal hydroxides and oxyhydroxides responsible for in situ coagulation:

4 $${\mathrm{Fe}}^{2+/3+}{+\mathrm{nH}}_{2}O\to \mathrm{Fe}{{\left(\mathrm{OH}\right)}_{n}}^{\left(\mathrm{3‐n}\right)}\left(s\right){+\mathrm{nH}}^{+}$$

At the cathode, water reduction produces hydrogen gas and hydroxide ions:

5 $${\mathrm{Cathode}:\;2H}_{2}{O+2e}^{‐}\to H_{2}\left(g\right){+\mathrm{2OH}}^{‐}$$

The generated hydroxides, mainly Fe­(OH)_2_ and Fe­(OH)_3_, act as highly efficient coagulants due to their high surface area and adsorption capacity. The overall mechanism of paracetamol removal can be described as follows: (i) generation of active metallic species; (ii) hydrolysis and formation of hydroxylated complexes; (iii) charge neutralization of contaminants and colloidal particles; (iv) aggregation and floc formation; (v) sedimentation via sweep coagulation; and (vi) flotation assisted by hydrogen bubbles generated at the cathode.

The 27-run FCCCD optimization was conducted with the regulated laboratory DC supply so that current density could be imposed as an experimental factor. The maximum-removal condition (Run 20) was subsequently assessed with the PV–MPPT–battery route. Because current was monitored but not actively controlled in the PV configuration, this PV observation was treated as a separate operating-scenario assessment and was not used to fit the FCCCD model or to claim a source-only causal effect.

PV-supplied experiments were carried out at the Universidade Tecnológica Federal do Paraná campus (UTFPR–Londrina, Brazil; 23°18′36.6″ S, 51°09′46.1″ W; approximate altitude 610 m).

### 2.4 Experimental Design and Statistical Analysis

The optimization of the electrocoagulation process using iron electrodes for paracetamol removal was carried out through Response Surface Methodology (RSM) using STATISTICA software (v. 12.0, StatSoft Inc., USA). This methodology integrates mathematical and statistical tools that enable the modeling of multivariable systems in which one or more independent factors influence a response variable, with the objective of identifying the optimal operating conditions.

RSM overcomes the limitations of conventional experimental approaches, in which the effect of a single factor is commonly evaluated while keeping the remaining factors constant, thereby neglecting potential interactions among variables. In contrast, RSM allows the simultaneous evaluation of linear, quadratic, and interaction effects between the investigated factors.^24^

The mathematical models correlating the investigated responses with the operational parameters were developed using second-order multiple regression analysis, according to eq 6 ^25,26^

6 $${R=b}_{o}+\overset{k}{\underset{i=1}{\sum }}b_{i}q_{i}+\overset{k}{\underset{i=1}{\sum }}b_{\mathrm{ii}}{q_{i}}^{2}+\overset{k‐1}{\underset{i=1}{\sum }}\overset{k}{\underset{j=i+1}{\sum }}b_{\mathrm{ij}}q_{i}q_{j}$$

where R represents the studied response (paracetamol removal efficiency (%) and final concentration), k is the number of operational variables, q_i_ represents the independent factors, and b_0_, b_i_, b_ii_, and b_ij_ are the intercept, linear, quadratic, and interaction regression coefficients, respectively.

Table 2 presents the independent operational variables selected for the experimental design, together with their corresponding coded levels and actual values. The factors considered were initial paracetamol concentration (q_1_), reaction time (q_2_), current density (q_3_), and initial pH (q_4_), defined according to their expected influence on coagulant species generation and the overall efficiency of the electrocoagulation process.

**Table 2:** Independent Factors and Coded Levels Used in the Face-Centered Central Composite Design for Electrocoagulation Optimization^a^

Independent variable	Symbol	Unit	–α	–1	0	+1	+α
Initial paracetamol concentration	q_1_	mg·mL^–1^	0.1	0.1	0.2	0.3	0.3
Reaction time	q_2_	min	10	10	20	30	30
Current density	q_3_	A·m^–2^	0.95	0.95	3.81	6.67	6.67
Initial pH	q_4_	–	3.0	3.0	6.5	10.0	10.0

a Since the selected design corresponds to a Face-Centered Central Composite Design (FCCCD) with α = 1, the axial levels (−α and +α) coincide with the factorial levels (−1 and +1), respectively. Consequently, all experimental runs remain within the predefined experimental domain

A Face-Centered Central Composite Design (FCCCD) was employed for experimental optimization. In this design, the axial points are located at α = ±1 on the faces of the factorial cube, so all runs remain within the predefined pH, concentration, time, and current-density limits.^27,28^ Compared with a circumscribed central composite design, this avoids axial conditions outside the feasible domain. The trade-off is that the FCCCD is generally nonrotatable and may have less uniform prediction variance away from the design center.

The experimental matrix generated through the FCCCD is summarized in Table 3. The design consisted of a total of 27 experimental runs, including 16 factorial points, 8 axial points, and 3 replicated center points. This experimental matrix served as the basis for fitting the quadratic model and for subsequent process optimization using Response Surface Methodology.

**Table 3:** Experimental Matrix of the Face-Centered Central Composite Design (FCCCD) Used for the Optimization of Paracetamol Removal by Electrocoagulation^a^

Run	q_1_: Initial paracetamol concentration (mg·mL^–1^)	q_2_: Reaction time (min)	q_3_: Current density (A·m^–2^)	q_4_: Initial pH (−)
1	0.1	10	0.95	3.0
2	0.3	10	0.95	3.0
3	0.1	30	0.95	3.0
4	0.3	30	0.95	3.0
5	0.1	10	6.67	3.0
6	0.3	10	6.67	3.0
7	0.1	30	6.67	3.0
8	0.3	30	6.67	3.0
9	0.1	10	0.95	10.0
10	0.3	10	0.95	10.0
11	0.1	30	0.95	10.0
12	0.3	30	0.95	10.0
13	0.1	10	6.67	10.0
14	0.3	10	6.67	10.0
15	0.1	30	6.67	10.0
16	0.3	30	6.67	10.0
17	0.1	20	3.81	6.5
18	0.3	20	3.81	6.5
19	0.2	10	3.81	6.5
20	0.2	30	3.81	6.5
21	0.2	20	0.95	6.5
22	0.2	20	6.67	6.5
23	0.2	20	3.81	3.0
24	0.2	20	3.81	10.0
25	0.2	20	3.81	6.5
26	0.2	20	3.81	6.5
27	0.2	20	3.81	6.5

a Runs 1–16 correspond to the factorial points, runs 17–24 represent the axial points, and runs 25–27 correspond to replicated center points. Center-point replicates were included to estimate experimental error and improve model reliability.

The experimental data were fitted to a second-order polynomial model containing linear, quadratic, and interaction terms. Model and coefficient significance was evaluated by analysis of variance (ANOVA) at a 95% confidence level (*p* < 0.05), following standard response-surface methodology and experimental-design guidance.^27,28^ Model adequacy was assessed using R^2^, adjusted R^2^, the F-statistic, and residual error; statistical significance was not equated with high predictive accuracy.

Three-dimensional response surface plots and contour diagrams were generated to visualize the combined effects of the investigated variables and to identify the optimal operating region for maximizing paracetamol removal efficiency. Additionally, zeta potential (ζ) and hydrodynamic diameter were evaluated as complementary response variables in order to correlate statistical optimization with the underlying colloidal mechanisms involved in the electrocoagulation process.^14,29^

Optimization was performed under the regulated conventional DC supply. The selected condition was then operated with the PV–MPPT–battery route as a separate feasibility scenario. PV supply was not included as an FCCCD factor, and the reactor electrical demand—not solar availability—was the quantity to be minimized before PV sizing.

### 2.5 Techno-Economic Evaluation

Energy use, theoretical iron dissolution, and partial variable cost

Specific reactor energy consumption (E, kWh·m^–3^) was calculated as

7 $$E=\frac{U\times I\times t_{h}}{1000\times V_{r}}$$

where U is the reactor-terminal voltage (V), I is the reactor current (A), t_h_ is the electrolysis time (h), and V_r_ is the treated volume (m^3^). The factor 1000 converts Wh to kWh.

Theoretical iron dissolution (mFe, kg·m^–3^) was estimated using Faraday’s law:

8 $$mFe=\frac{I\times t_{s}\times MFe}{z\times F\times V_{r}}$$

where I is the measured reactor current (A), t_s_ is the electrolysis time (s), mFe = 0.05585 kg·mol^–1^, z = 2, F = 96 485 C·mol^–1^, and V_r_ is the treated volume (m^3^).

The partial variable cost (C_partial_, BRL·m^–3^) was calculated as

9 $$C_{\mathrm{partial}}=\left(\mathrm{aE}\right)+\left(\mathrm{bmFe}\right)$$

where a is the electricity purchase price (BRL·kWh^–1^) and b is the iron unit price (BRL·kg^–1^). For the focused Run 20 comparison, the calculation used agrid = BRL 0.7091·kWh^–1^, aPV = BRL 0.0000·kWh^–1^ under the marginal-cost assumption, and b = BRL 0.285·kg^–1^. These values are scenario inputs and should not be interpreted as a complete life-cycle electricity or treatment cost.

The reported operational cost is a partial variable cost containing only the electricity and theoretical iron-consumption terms in eq 9 PV module and controller capital, battery replacement, financing, electrode cleaning or replacement, sludge handling, labor, and life-cycle environmental impacts were excluded; therefore, the values should not be interpreted as total treatment cost.

For the focused comparison at Run 20, the same reactor energy consumption and theoretical iron dissolution were used for the conventional and photovoltaic configurations. Therefore, the difference in partial variable cost arises exclusively from the electricity purchase price assigned to each scenario and not from a change in reactor energy demand, iron dissolution, or electrocoagulation performance.

## 3 Results and Discussion

### 3.1 Experimental Results

Table 4 presents the experimental results obtained for paracetamol removal by electrocoagulation using iron electrodes under the operational conditions established by the Face-Centered Central Composite Design (FCCCD). For each experimental run, the values of the independent operational variables, final paracetamol concentration, and corresponding removal efficiency are reported. These data constituted the basis for fitting the quadratic model and for subsequent statistical analysis using Response Surface Methodology (RSM).

**Table 4:** Experimental Results of Paracetamol Removal by Electrocoagulation under the Conditions of the Face-Centered Central Composite Design (FCCCD)^a^

Run	q_1_: Initial paracetamol concentration (mg·mL^–1^)	q_2_: Reaction time (min)	q_3_: Current density (A·m^–2^)	q_4_: Initial pH	Final paracetamol concentration (mg·mL^–1^)	Removal (%)
1	0.1	10	0.95	3.0	0.033	67.0
2	0.3	10	0.95	3.0	0.087	71.0
3	0.1	30	0.95	3.0	0.027	73.0
4	0.3	30	0.95	3.0	0.198	34.0
5	0.1	10	6.67	3.0	0.0237	76.3
6	0.3	10	6.67	3.0	0.066	78.0
7	0.1	30	6.67	3.0	0.0237	76.3
8	0.3	30	6.67	3.0	0.071	76.3
9	0.1	10	0.95	10.0	0.0355	64.5
10	0.3	10	0.95	10.0	0.099	67.0
11	0.1	30	0.95	10.0	0.027	73.0
12	0.3	30	0.95	10.0	0.090	70.0
13	0.1	10	6.67	10.0	0.071	29.0
14	0.3	10	6.67	10.0	0.066	78.0
15	0.1	30	6.67	10.0	0.022	78.0
16	0.3	30	6.67	10.0	0.072	76.0
17	0.1	20	3.81	6.5	0.024	76.0
18	0.3	20	3.81	6.5	0.099	67.0
19	0.2	10	3.81	6.5	0.064	68.0
20	0.2	30	3.81	6.5	0.0186	90.7
21	0.2	20	0.95	6.5	0.026	87.0
22	0.2	20	6.67	6.5	0.065	67.5
23	0.2	20	3.81	3.0	0.070	65.0
24	0.2	20	3.81	10.0	0.065	67.5
25	0.2	20	3.81	6.5	0.070	65.0
26	0.2	20	3.81	6.5	0.070	65.0
27	0.2	20	3.81	6.5	0.070	65.0

a Note: Removal efficiency was calculated using eq 2. Final paracetamol concentrations are reported with up to four decimal places when required to preserve numerical consistency between concentration and removal efficiency.

The highest paracetamol removal efficiency (90.7%) was achieved under operational conditions corresponding to an initial paracetamol concentration of 0.2 mg·mL^–1^, a reaction time of 30 min, a current density of 3.81 A·m^–2^, and an initial pH of 6.5 (Run 20). In contrast, the lowest efficiency (29.0%) was observed at 0.1 mg·mL^–1^, 10 min, 6.67 A·m^–2^, and pH 10.0 (Run 13).

Overall, intermediate current densities (3.81 A·m^–2^) and near-neutral pH conditions (pH ≈ 6.5) favored process performance, resulting in removal efficiencies exceeding 85–90%. This behavior may be attributed to the formation of amorphous iron hydroxide species with high adsorption capacity, which enhance coagulation, adsorption, and coprecipitation mechanisms involved in contaminant removal.

Likewise, reaction times around 20–30 min promoted a more favorable system performance, whereas shorter reaction times resulted in moderate efficiencies (65–78%), possibly due to initial kinetic limitations or stabilization phenomena occurring within the electrochemical system.

Taken together, these results indicate the multivariable behavior of the electrocoagulation process, in which treatment performance depended on the coupled effects of initial paracetamol concentration, reaction time, current density, and initial pH.^30–32^ Because these variables produced nonlinear and interaction effects, Response Surface Methodology provided a framework for identifying a favorable operating region within the investigated domain.^33^

### 3.2 Statistical Modeling and Optimization (RSM)

#### 3.2.1 Statistical Modeling and Interpretation of Regression Coefficients

Table 5 summarizes the estimated effects and regression coefficients of the second-order polynomial model developed through Response Surface Methodology (RSM) to describe paracetamol removal by electrocoagulation using iron electrodes.

**Table 5:** Estimated Effects and Regression Coefficients of the Quadratic Model Developed through Response Surface Methodology (RSM) for Paracetamol Removal (%)^a^ ^b^

Model term	Effect	SE (Effect)	T	p-value	Coefficient	SE (Coeff.)	95% CI (Coeff.)
Intercept	65.0000	5.0791	12.7975	<0.0001	65.0000	5.0791	53.9335 to 76.0665
Concentration (L) (mg·mL^–1^)	–2.8750	3.5915	–0.8005	0.4390	–1.4375	1.7957	–5.3501 to 2.4751
Concentration (Q)	1.3785	3.8093	0.3619	0.7237	0.6892	1.9047	–3.4607 to 4.8392
Time (L) (min)	7.8194	3.5915	2.1772	0.0501	3.9097	1.7957	–0.0029 to 7.8223
Time (Q)	5.2951	3.8093	1.3900	0.1898	2.6476	1.9047	–1.5024 to 6.7975
Current density (L) (A·m^–2^)	–1.0972	3.5915	–0.3055	0.7652	–0.5486	1.7957	–4.4612 to 3.3640
Current density (Q)	4.2535	3.8093	1.1166	0.2860	2.1267	1.9047	–2.0232 to 6.2767
Initial pH (L)	1.5139	3.5915	0.4215	0.6808	0.7569	1.7957	–3.1556 to 4.6695
Initial pH (Q)	–1.2465	3.8093	–0.3272	0.7491	–0.6233	1.9047	–4.7732 to 3.5267
Concentration × Time	–9.4375	4.3987	–2.1455	0.0531	–4.7188	2.1993	–9.5107 to 0.0732
Concentration × Current density	11.3958	4.3987	2.5908	0.0236	5.6979	2.1993	0.9060 to 10.4898
Concentration × pH	9.9792	4.3987	2.2687	0.0425	4.9896	2.1993	0.1977 to 9.7815
Time × Current density	8.1042	4.3987	1.8424	0.0902	4.0521	2.1993	–0.7398 to 8.8440
Time × pH	10.5208	4.3987	2.3918	0.0340	5.2604	2.1993	0.4685 to 10.0523
Current density × pH	–12.6458	4.3987	–2.8749	0.0140	–6.3229	2.1993	–11.1148 to – 1.5310

a Face-Centered Central Composite Design (FCCCD; 4 factors, n = 27; residual mean square, MS = 77.3926; R^2^ = 0.7818).

b Note: L and Q represent linear and quadratic effects, respectively. Terms with p < 0.05 were considered statistically significant at the 95% confidence level.

The obtained model showed overall statistical significance and explained 78.18% of the experimental variability of the response (R^2^ = 0.7818). However, after accounting for the number of regression terms included in the model, the adjusted coefficient of determination was lower (adjusted R^2^ = 0.5273), indicating that the full quadratic model exhibited a moderate explanatory capability. The root-mean-square error (RMSE) was 8.80%, representing the average residual deviation between experimental and predicted values in terms of removal percentage.

This behavior can be considered reasonable for electrocoagulation systems, which are governed by multiple simultaneous physicochemical phenomena, including anodic iron dissolution, Fe^2+^/Fe^3+^ hydrolysis, formation of amorphous iron hydroxides, colloidal destabilization, floc growth, hydrogen evolution, and possible secondary reactions occurring in the electrochemical medium. Consequently, the model should primarily be interpreted as a tool for identifying dominant trends and optimizing the operational region within the investigated experimental domain, rather than as a universal predictive equation for the process.

Regression analysis revealed that the individual linear and quadratic effects associated with initial paracetamol concentration, reaction time, current density, and initial pH did not exhibit statistical dominance at the 95% confidence level (*p* > 0.05). Nevertheless, the linear effect of reaction time showed a value close to the significance threshold (p = 0.0501), suggesting a marginal contribution to paracetamol removal efficiency.

Similarly, the interaction between initial concentration and reaction time (q1 × q2) exhibited a near-significant effect (p = 0.0531), suggesting a possible secondary contribution to process performance that should be interpreted cautiously from both statistical and mechanistic perspectives.

In contrast, the response was mainly governed by interaction terms. The interactions concentration × current density (p = 0.0236), concentration × pH (p = 0.0425), time × pH (p = 0.0340), and current density × pH (p = 0.0140) were statistically significant. This indicates that paracetamol removal was not controlled by isolated variables, but by the coupled effects of pollutant load, electrochemical coagulant generation, reaction time, and solution chemistry.

Among the significant terms, the current density × pH interaction presented the strongest statistical contribution and a negative coefficient. This antagonistic effect suggests that increasing current density does not necessarily improve removal efficiency under all pH conditions. From a mechanistic perspective, current density controls the rate of iron dissolution and the generation of coagulant species, whereas pH governs Fe^2+^/Fe^3+^ hydrolysis, Fe­(OH)­x formation, and the surface charge of colloidal aggregates. Under near-neutral conditions, amorphous iron hydroxides are favored, promoting charge neutralization, adsorption, and sweep coagulation. Conversely, under strongly acidic or alkaline conditions, or under an unbalanced electrochemical dose, less effective iron species, enhanced hydrogen evolution, or precipitates with lower adsorption capacity may reduce process performance.

Overall, the results indicate that the evaluated response depended on simultaneous electrochemical and chemical conditions. The RSM model was used to identify favorable operating regions and interaction terms within the experimental domain; its adjusted R^2^ = 0.5273 limits claims of predictive robustness.

#### 3.2.2 Pareto Chart of Standardized Effects for Paracetamol Removal by Iron Electrocoagulation

Figure 2 presents the Pareto chart of standardized effects, illustrating the relative influence of operational variables on paracetamol removal efficiency as obtained from the response surface methodology model. The vertical dashed line represents the critical t-value at a 95% confidence level; effects exceeding this threshold are considered statistically significant.

**Figure 2:**
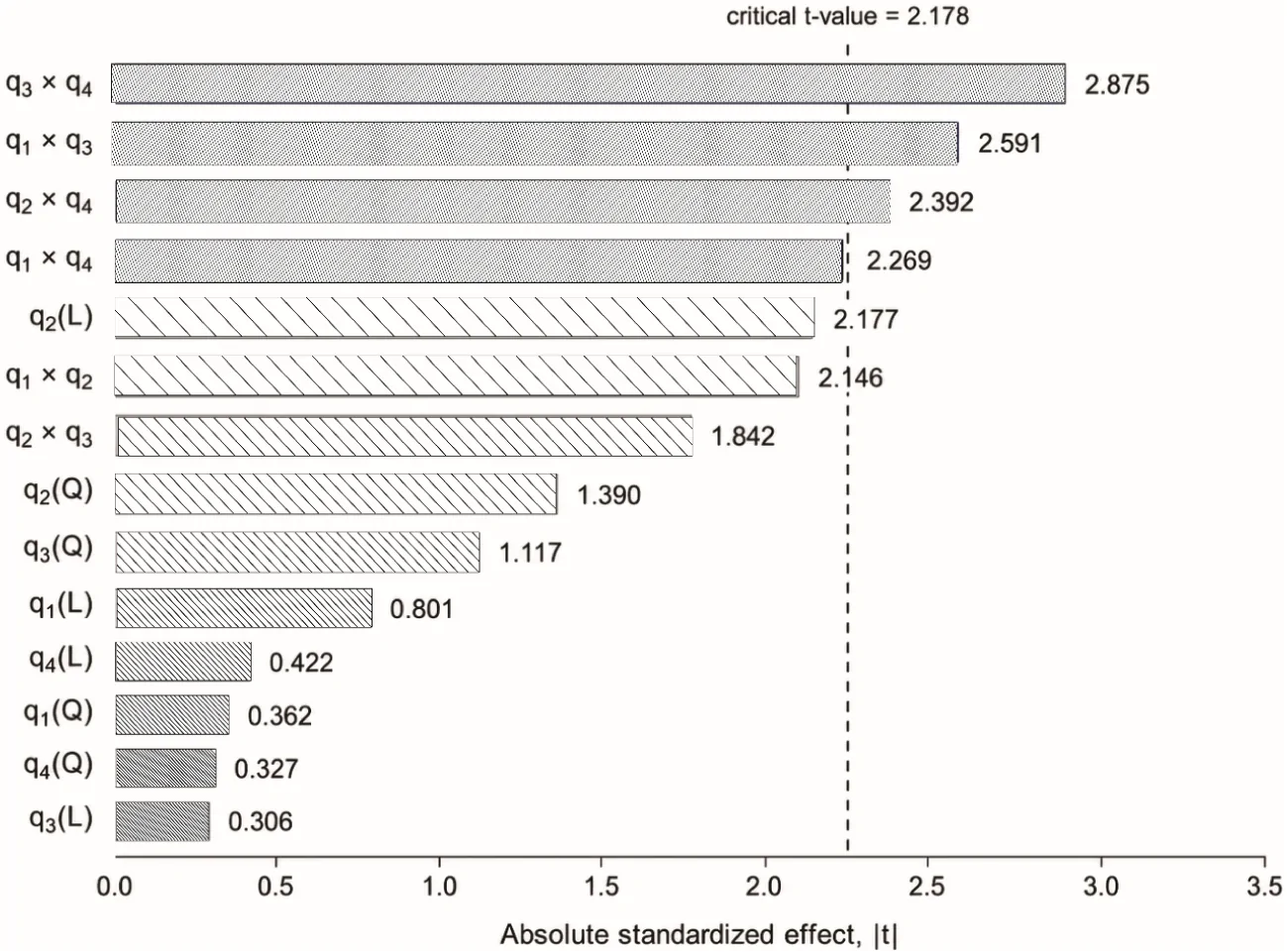
Pareto chart of standardized effects for paracetamol removal (%) obtained from the response surface methodology model. The dashed line represents the critical t-value at a 95% confidence level. Terms exceeding this threshold are considered statistically significant, highlighting the interactions q_3_ × q_4_, q_1_ × q_3_, q_2_ × q_4_, and q_1_ × q_4_, which confirm the nonlinear and multivariable nature of the electrocoagulation process.

The results indicate that the interaction q_3_ × q_4_ (current density × initial pH) exhibits the highest standardized effect (|t| ≈ 2.87), highlighting the strong coupling between the electrochemical generation of iron hydroxide species and the solution chemistry governing their speciation. Similarly, the interactions q_1_ × q_3_ (initial concentration × current density), q_2_ × q_4_ (reaction time × pH), and q_1_ × q_4_ (initial concentration × pH) also exceed the statistical significance threshold, demonstrating that the electrocoagulation process is primarily governed by synergistic interactions between electrochemical and physicochemical variables.

In contrast, the individual linear and quadratic effects of the factors (q_1_, q_2_, q_3_, and q_4_) exhibit comparatively lower standardized contributions. These results further confirm the multivariable and nonlinear nature of the electrocoagulation system, in which process performance is not governed by isolated parameters, but rather by the combined influence of current density, reaction time, contaminant concentration, and pH.

#### 3.2.3 Statistical Validation and Model Limitations

Table 6 presents the statistical validation of the quadratic model obtained through Response Surface Methodology (RSM), performed using analysis of variance (ANOVA), Pareto chart analysis of standardized effects, and comparison between observed and predicted values.

**Table 6:** ANOVA of the Quadratic Model (RSM) for Paracetamol Removal (%)^a^

Source	df	SS (Sum of Squares)	MS (Mean Square)	F	p-value
Model (Regression)	14	3328.305	237.736	3.072	0.0293
Concentration (L) (mg·mL^–1^)	1	49.593	49.593	0.641	0.4390
Concentration (Q)	1	10.135	10.135	0.131	0.7237
Time (L) (min)	1	366.863	366.863	4.740	0.0501
Time (Q)	1	149.539	149.539	1.932	0.1898
Current density (L) (A·m^–2^)	1	7.224	7.224	0.093	0.7652
Current density (Q)	1	96.472	96.472	1.247	0.2860
Initial pH (L)	1	13.762	13.762	0.178	0.6808
Initial pH (Q)	1	8.286	8.286	0.107	0.7491
Concentration × Time	1	356.421	356.421	4.603	0.0531
Concentration × Current density	1	519.518	519.518	6.712	0.0236
Concentration × pH	1	398.561	398.561	5.146	0.0425
Time × Current density	1	262.742	262.742	3.395	0.0902
Time × pH	1	442.691	442.691	5.721	0.0340
Current density × pH	1	639.664	639.664	8.266	0.0140
Error (Residual)	12	928.711	77.393	—	—
Total	26	4257.016	—	—	—

a Face-Centered Central Composite Design (FCCCD) (4 factors; n = 27; df_total = 26; df_model = 14; df_error = 12; MS_error = 77.3926; R^2^ = 0.7818).

The overall model was statistically significant (F = 3.072; p = 0.0293), with a coefficient of determination R^2^ = 0.7818, indicating that approximately 78% of the experimental variability in paracetamol removal was explained by the regression model.

The residual mean square (MS = 77.39) is consistent with the expected variability in electrocoagulation processes, which are characterized by the coupling of electrochemical kinetics, iron speciation, and particle aggregation phenomena.

The ANOVA breakdown indicates that individual linear terms (concentration, time, current density, and pH) are not the primary determinants of the process (*p* > 0.05 in most cases). In contrast, several second-order interactions were statistically significant, confirming the coupled nature of the system. In particular, the interaction current density × pH (p = 0.0140) exhibited the highest absolute effect and a negative coefficient, indicating antagonistic behavior under extreme combinations of these variables.

This behavior can be explained by the strong dependence of Fe^2+^/Fe^3+^ speciation on pH, which governs the formation of Fe­(OH)_3_ and active polymeric species. At high current densities and unfavorable pH conditions, parallel reactions such as hydrogen evolution may be intensified, and finer precipitates with lower adsorption capacity may be generated, thereby reducing the overall process efficiency.

Similarly, the interactions concentration × current density (p = 0.0236), concentration × pH (p = 0.0425), and time × pH (p = 0.0340) were also significant, confirming that removal efficiency is governed by the balance between coagulant generation, pollutant load, and the chemical environment of the solution. This nonadditive behavior is consistent with electrocoagulation systems, where the effective electrochemical dose depends simultaneously on the applied current density and the chemical regime imposed by pH.

The Pareto chart shows that these interaction terms exceeded the selected significance threshold, whereas individual main effects had smaller standardized contributions. This pattern is consistent with coupled electrochemical–chemical effects but does not, by itself, establish a mechanistic pathway.

Finally, Figure 3 presents the comparison between the experimental and predicted paracetamol removal efficiencies for the 27 experimental runs. Overall, the data points are distributed close to the identity line (y = x), indicating acceptable agreement between the experimentally obtained values and those estimated by the quadratic RSM model. This behavior is consistent with the R^2^ value of 0.7818, indicating that the model explained approximately 78.2% of the experimental variability in the response.

**Figure 3:**
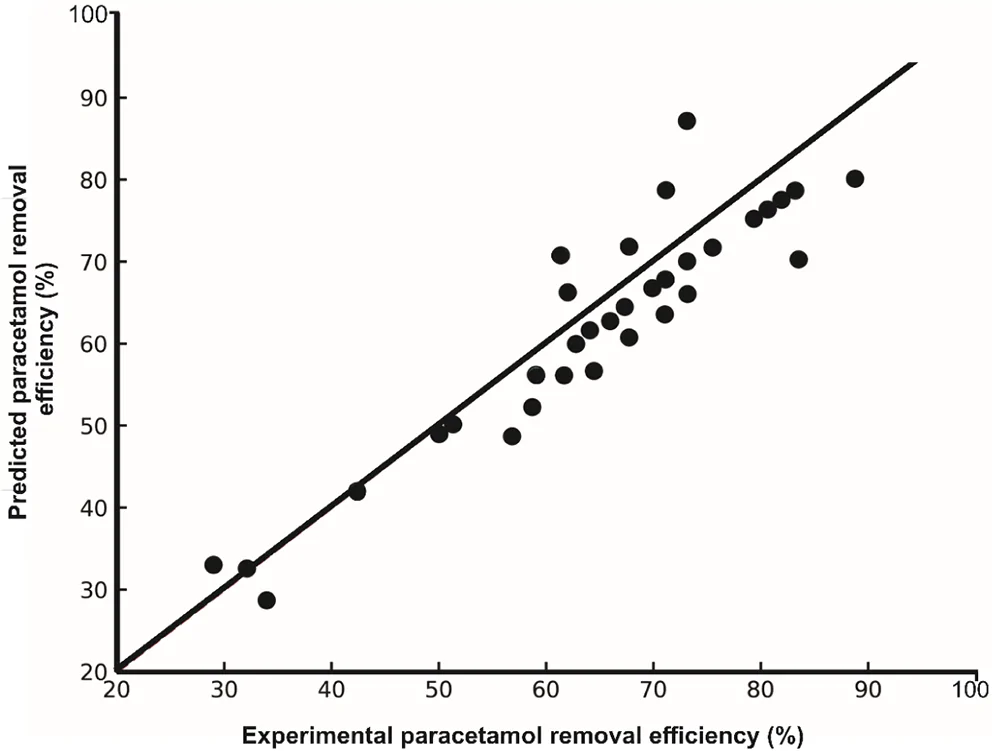
Comparison between the experimental and predicted paracetamol removal efficiency (%) obtained using the quadratic RSM model. The dashed line represents the identity line (y = x), corresponding to perfect agreement between the experimental and predicted values.

Most of the data were concentrated within the intermediate removal range, approximately between 65 and 80%, where the model adequately reproduced the central trend of the process. However, more pronounced deviations were observed at the extremes of the experimental domain. In the low-removal region, with experimental values close to 29–35%, the model tended to slightly overestimate the response. Conversely, in the high-efficiency region, with experimental removals close to 83–91%, some isolated deviations were observed, suggesting lower predictive accuracy under boundary conditions.

This behavior is consistent with the empirical nature of quadratic models applied to multivariable electrochemical systems, where the measured response may be affected by localized phenomena that are not fully represented by the fitted response surface. In electrocoagulation, these effects may include changes in iron speciation and hydrolysis equilibria, local pH gradients near the electrode surface, hydrogen-bubble generation, floc nucleation and growth, and progressive modifications of the electrode surface.^34–37^ Therefore, the fitted model should be interpreted primarily as an optimization and trend-identification tool within the evaluated experimental domain, rather than as a universal predictive equation for operating conditions outside the studied range.^38^

Table 7 summarizes the RSM model fitted to the regulated-supply iron electrocoagulation experiments. The quadratic model was statistically significant (F = 3.072; p = 0.0293) and explained 78.18% of the observed variability. The adjusted R^2^ of 0.5273 indicates moderate explanatory capacity; therefore, the model is used for trend identification within the tested domain rather than as evidence of universal predictive performance.

**Table 7:** Statistical Summary of the Iron Electrocoagulation Model^a^

Parameter	Value	Relevance
Experimental design	FCCCD, 4 factors, 27 runs	Suitable for evaluating linear, quadratic, and interaction effects.
Model type	Quadratic RSM model	Used to describe the nonlinear behavior of paracetamol removal.
F-value	3.072	Indicates overall model significance.
p-value	0.0293	The model was statistically significant at *p* < 0.05.
R^2^	0.7818	The model explained 78.18% of the experimental variability.
Adjusted R^2^	0.5273	Indicates moderate explanatory capacity after accounting for model complexity.
RMSE	8.80%	Average residual deviation in removal efficiency units.
Most significant term	Current density × pH	Strongest interaction effect; *p* = 0.0140.
Dominant model behavior	Interaction-driven	Removal was mainly governed by combined effects rather than isolated variables.

a The principal model statistics were obtained from the FCCCD-RSM analysis: 27 runs, a quadratic model, F = 3.072, p = 0.0293, R^2^ = 0.7818, residual mean square = 77.3926, and interaction terms led by current density × pH.

### 3.3 Response Surface Methodology: Analysis of Paracetamol Removal (%)

Figure 4 presents the three-dimensional response surfaces, together with their contour projections, for paracetamol removal efficiency (%) as a function of binary combinations of the operational variables of the electrocoagulation (EC) reactor. These surfaces allow the simultaneous interpretation of curvature (quadratic effects) and interactions (cross effects), and help identify operational regions in which the system maximizes removal efficiency without incurring unnecessary energy overconsumption.

**Figure 4:**
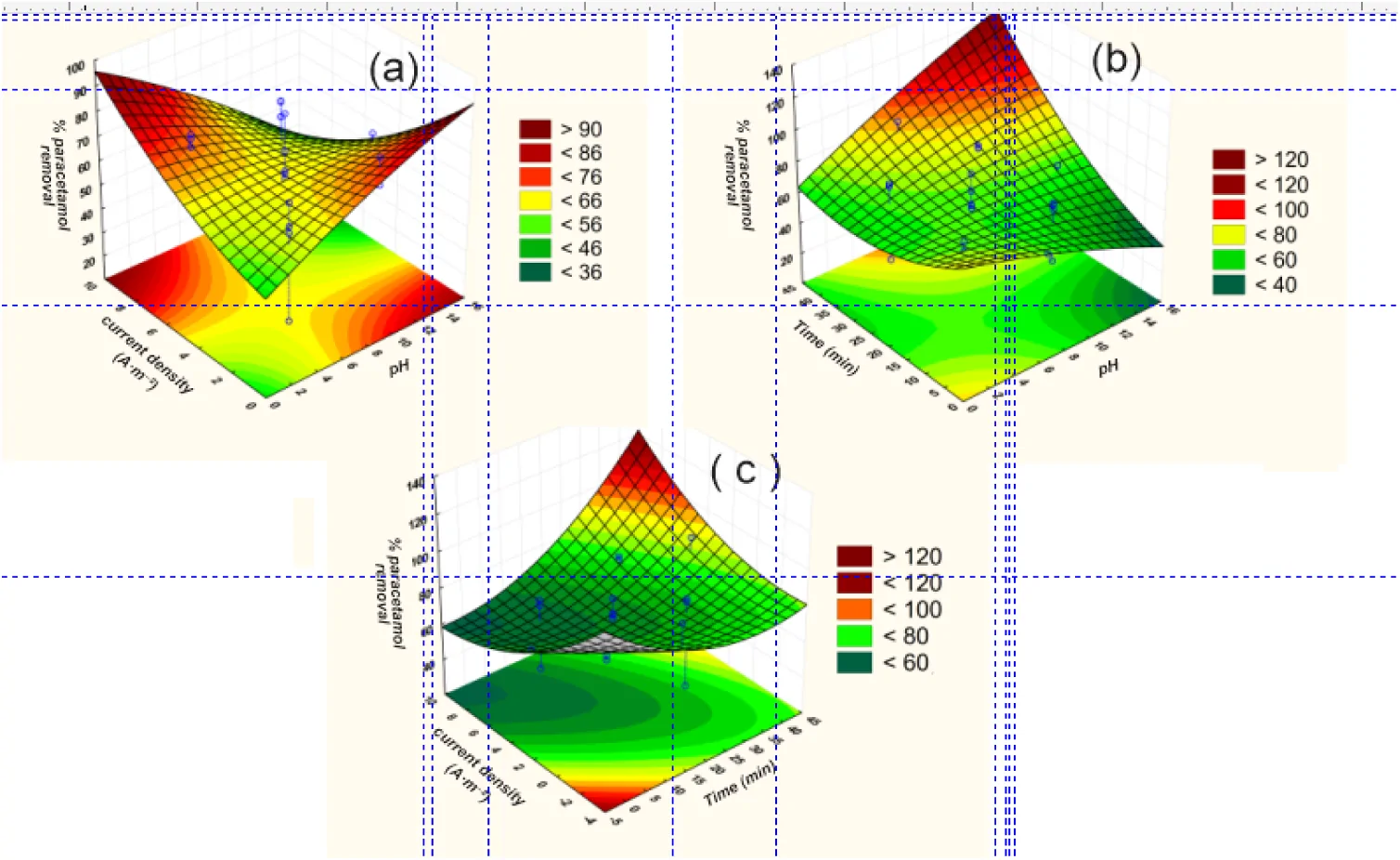
Three-dimensional response surface plots (RSM) for paracetamol removal (%): (a) current density–initial pH, (b) time–initial pH, and (c) current density–time. Experimental data points are superimposed on the fitted quadratic model, highlighting the optimal operating region within the evaluated experimental domain.

#### 3.3.1 Current Density–pH Interaction

The current density–pH response surface (Figure 4a) revealed a marked interaction between both variables. Removal efficiency remained moderate, approximately 40–50%, under strongly acidic conditions around pH 3, but increased to values above 90% in the near-neutral range of pH 6–7 when the current density was maintained between 4 and 6 A m^–2^. This enhancement can be attributed to the favorable formation of iron hydrolysis products and hydroxide/oxyhydroxide flocs within this pH interval.35,39 At alkaline pH values above 9, the removal efficiency decreased again to approximately 60–70%, even at higher current densities, indicating that excessive alkalinity may alter the dominant iron species and reduce the effectiveness of the active coagulant forms.^39,40^ The steepest response gradient was observed between pH 4 and 7, where removal efficiency increased by approximately 30–40 percentage points, confirming that pH and current density act as coupled control variables rather than independent factors in the electrocoagulation process.^41–43^

#### 3.3.2 Time–pH Interaction

The time–pH surface (Figure 4b) shows that increasing reaction time from 10 to 30 min enhances removal efficiency from approximately 60% to 85–90%, provided that pH remains near neutrality. However, under acidic conditions near the lower boundary of the evaluated range (around pH 3–4), removal efficiency remains limited below 60%, regardless of reaction time. Therefore, the effect of time is pH-dependent, with a maximum increase of approximately 25–30 percentage points within the optimal operating window

#### 3.3.3 Current Density–Time Interaction

In the current density–time interaction (Figure 4c), removal efficiency increases progressively from values below 50% (1 A·m^–2^, 10 min) to above 85% when the current density reaches 5–6 A·m^–2^ and the reaction time approaches 30 min. However, the surface shows a plateau tendency in the upper region, indicating that further increases in current density or reaction time within the evaluated domain result in only marginal improvements (<5%). This suggests the existence of an optimal electrochemical dose, beyond which additional energy input does not translate into significant removal gains.

### 3.4 Response Surface Methodology: Final Paracetamol Concentration

Figure 5 presents the response surface plots (RSM) for the final paracetamol concentration (mg·mL^–1^) after electrocoagulation using iron electrodes. The interactions between current density–initial concentration (a), pH–initial concentration (b), and time–initial concentration (c) are shown, while the remaining variables were kept constant at their central levels. The predicted values range approximately from 0.01 to 0.09 mg·mL^–1^, indicating a nonlinear behavior governed by the electrochemical dose and iron speciation.

**Figure 5:**
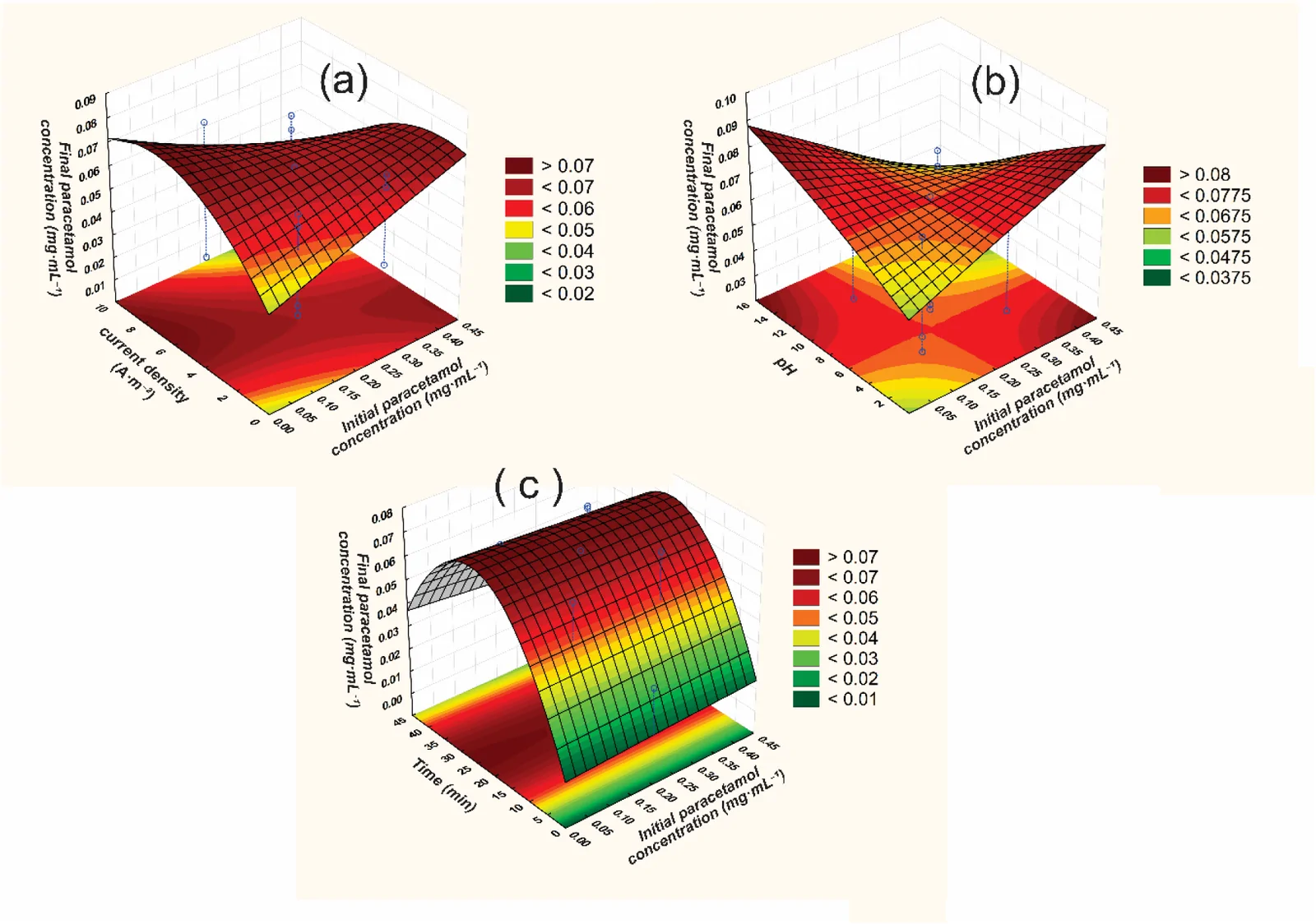
Response surface plots for final paracetamol concentration (mg·mL^–1^): (a) current density–initial concentration, (b) pH–initial concentration, and (c) time–initial concentration interactions. Experimental points are superimposed on the fitted quadratic model surfaces.

#### 3.4.1 Current Density–Initial Concentration Interaction

The current density–initial concentration interaction (Figure 5a) showed that, at low current density (1 A m^–2^), the residual paracetamol concentration remained relatively high, ranging from approximately 0.06 to 0.08 mg mL^–1^, even at the lowest initial concentration evaluated (0.10 mg mL^–1^). However, when the current density increased to 5–6 A m^–2^, the residual concentration decreased to approximately 0.02–0.03 mg mL^–1^, particularly within the intermediate-to-high initial concentration range (0.20–0.30 mg mL^–1^). This trend suggests that, as the pollutant load increases, the generation rate of electrochemically produced iron coagulant species becomes a controlling factor for removal efficiency.^42–44^ Thus, increasing the current density enhances anodic dissolution and promotes the formation of iron hydroxide flocs, leading to reductions of up to approximately 60–70% in the residual paracetamol concentration.^44,45^

#### 3.4.2 pH–Initial Concentration Interaction

The pH–initial concentration surface (Figure 5b) reveals a strong dependence of the final concentration on iron speciation. Under acidic conditions near the lower boundary of the evaluated range (around pH 3–4), the residual concentration exceeds 0.07–0.08 mg·mL^–1^, regardless of the initial concentration. In contrast, within the pH range of 6–7, the final concentration decreases to approximatel y 0.03–0.04 mg·mL^–1^, representing a reduction of about 50% compared to extreme conditions.

At alkaline pH (>9), an increase in residual concentration (0.06–0.08 mg·mL^–1^) is observed again, confirming the existence of an optimal operating window near neutral pH.

#### 3.4.3 Time–Initial Concentration Interaction

In the time–initial concentration interaction (Figure 5c), short reaction times (10 min) produced residual concentrations of 0.06–0.08 mg·mL^–1^. As reaction time increased to 30 min, the concentration decreased to 0.02–0.03 mg·mL^–1^ for moderate initial concentrations. The modeled decrease was approximately 0.04–0.05 mg·mL^–1^. The surface then approached a plateau near the upper limit of the evaluated reaction-time range, where additional increases produced only marginal changes.

### 3.5 Colloidal Characterization before, during, and after Electrocoagulation

#### 3.5.1 Initial Colloidal Characterization before Electrocoagulation

Figure 6 shows the distribution of zeta potential and hydrodynamic diameter of the system prior to the electrocoagulation process. These measurements establish the initial colloidal state of the effluent and its electrostatic stability before the in situ generation of iron species.

**Figure 6:**
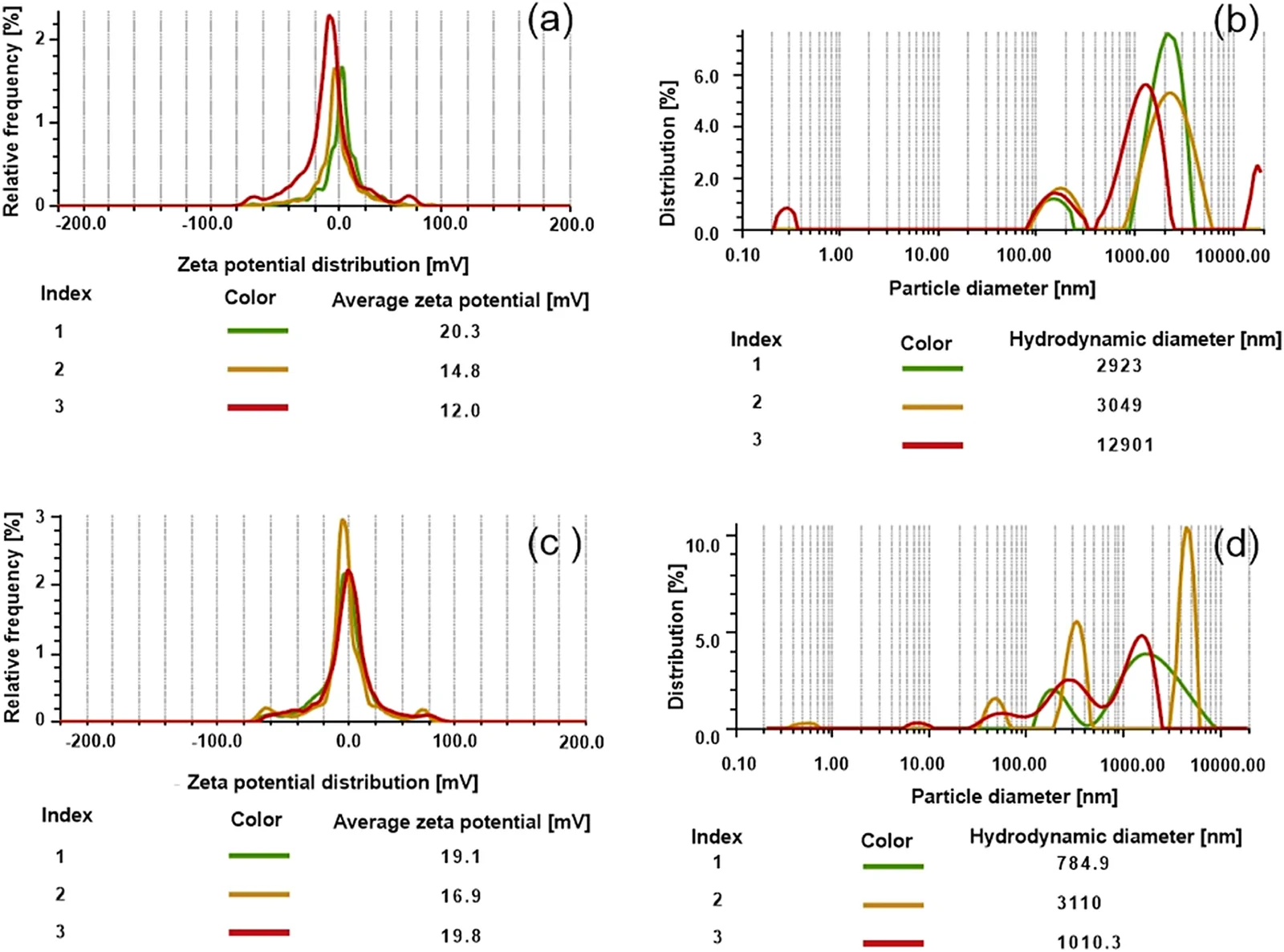
Initial colloidal characterization prior to electrocoagulation: (a) zeta potential distribution; (b) hydrodynamic diameter distribution; (c) zeta potential distribution; and (d) hydrodynamic diameter distribution.

In Figure 6a, the average zeta potential values were +20.3, + 14.8, and +12.0 mV, with distributions centered close to 0 mV but clearly shifted toward the positive region. These values suggest a colloidal system with moderate electrostatic stabilization, since zeta potentials in this range are generally insufficient to ensure strong colloidal stability but still indicate the presence of repulsive interactions capable of delaying rapid aggregation.^46,47^ The relatively narrow distributions further indicate the predominance of a particle population with comparatively homogeneous positive surface charge.

Figure 6b shows average hydrodynamic diameters of 2923, 3049, and 12901 nm, indicating micrometric aggregates and, in the latter case, a fraction above 10 μm. The coexistence of particles near 3 μm with larger aggregates is consistent with partial aggregation before treatment, possibly influenced by solute–particle interactions and ionic strength.^48–50^ The measured ζ-potential nevertheless indicates residual electrostatic repulsion before electrocoagulation.

In Figure 6c, the average zeta potential values were +19.1, +16.9, and +19.8 mV, which were slightly higher and more consistent than those observed in the previous system. This behavior indicates a more uniform colloidal condition, with lower surface-charge dispersion. The proximity of the values to approximately +20 mV suggests the presence of a relevant electrostatic barrier that limits spontaneous coalescence.

Figure 6d reveals hydrodynamic diameters of 784.9, 3110, and 1010.3 nm, indicating a bimodal size distribution composed of submicrometric fractions, approximately 0.8–1.0 μm, and micrometric aggregates close to 3 μm. Compared with the previous system, the lower formation of macroaggregates larger than 10 μm is consistent with the higher and more uniform zeta potential values, which reflect greater initial colloidal stability.^51^

#### 3.5.2 Evolution of the Colloidal State during Electrocoagulation

Figure 7 shows the evolution of zeta potential and hydrodynamic diameter during electrocoagulation. Relative to the initial state, surface-potential magnitude decreased and the particle-size distribution shifted as iron species were generated in situ.

**Figure 7:**
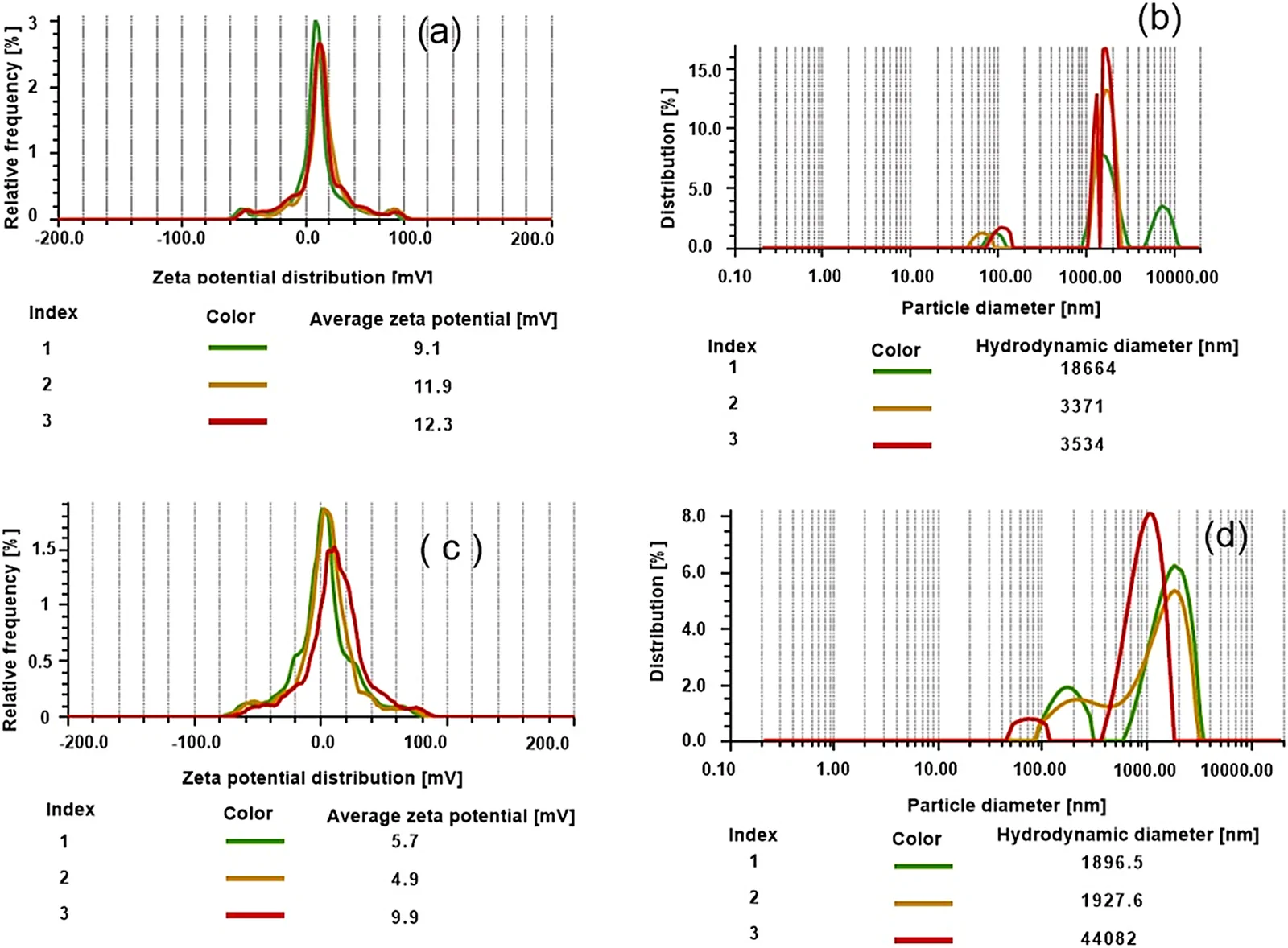
Colloidal characterization during electrocoagulation: (a) zeta potential distribution; (b) hydrodynamic diameter distribution; (c) zeta potential distribution; and (d) hydrodynamic diameter distribution.

In Figure 7a, the average zeta potential decreased to +9.1, + 11.9, and +12.3 mV, approximately 40–55% lower in magnitude than the initial values near +20 mV. The decrease is consistent with partial electrostatic neutralization, while the remaining positive values indicate residual repulsion. The narrower distribution around 0 mV is also consistent with a reduced interparticle repulsion barrier.

Figure 7b shows hydrodynamic diameters of 1866, 3371, and 3534 nm, indicating floc growth compared to the initial submicrometric fractions. The disappearance of extreme macroaggregates (>10 μm) and consolidation within the 2–3.5 μm range suggest the formation of more homogeneous and compact flocs. This behavior is consistent with a mechanism dominated by charge neutralization and sweep coagulation.

In Figure 7c, the average zeta potential decreased further to +5.7, + 4.9, and +9.9 mV. Values near +5 mV indicate a lower electrostatic-repulsion magnitude and conditions favorable to aggregation through particle collisions. Relative to the initial state, the magnitude decreased by approximately 70–75%.

Figure 7d shows average hydrodynamic diameters of 1896, 1927.6, and 44082 nm. The approximately 44 μm value is consistent with macrofloc formation and with sweep-coagulation or entrapment processes, but particle size alone does not identify a unique mechanism.

Overall, the measurements show a transition from ζ ≈ +20 mV toward a near-isoelectric regime accompanied by particle growth from the micrometric range to aggregates above 40 μm. This evolution is consistent with charge destabilization followed by floc growth; however, the ζ-potential and hydrodynamic-diameter measurements do not distinguish uniquely among adsorption, charge neutralization, and sweep-coagulation contributions.

#### 3.5.3 Colloidal State after Electrocoagulation

Figure 8 shows the distribution of zeta potential and hydrodynamic diameter after completion of the electrocoagulation process. The results indicate a transition toward conditions close to the isoelectric point and consolidation of floc size, confirming the structural stabilization of the formed aggregates.

**Figure 8:**
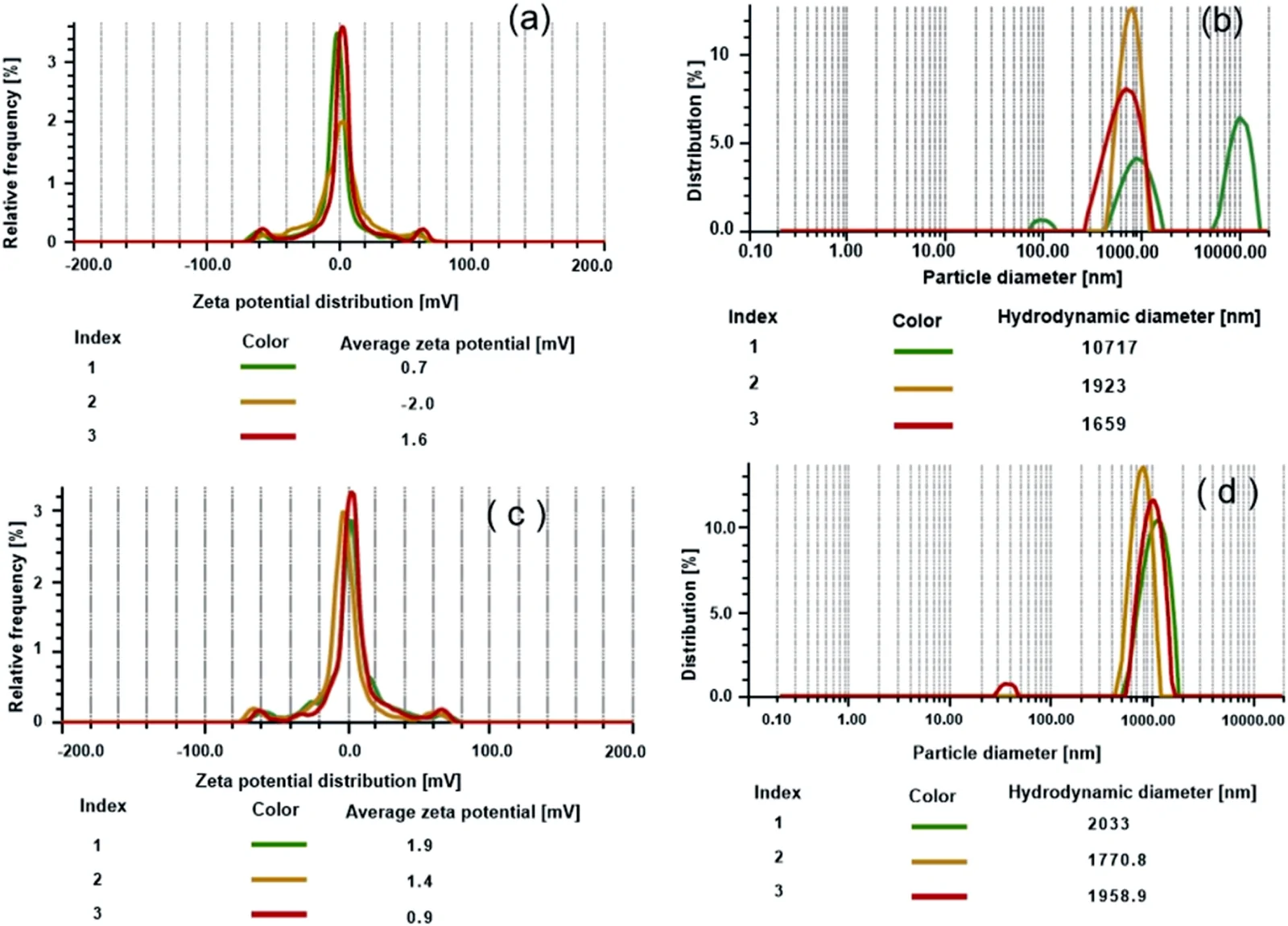
Colloidal characterization after electrocoagulation: (a) zeta potential distribution; (b) hydrodynamic diameter distribution; (c) zeta potential distribution; and (d) hydrodynamic diameter distribution.

In Figure 8a, the average zeta potential reaches values of +0.7 mV, – 2.0 mV, and +1.6 mV, indicating near-complete neutralization of surface charge. Compared to the initial state (+20 mV), this represents a reduction exceeding 90% in magnitude, placing the system under conditions close to the isoelectric point. This condition minimizes electrostatic repulsion and promotes irreversible aggregation.

Figure 8b shows average hydrodynamic diameters of 10717, 1923, and 1659 nm. The value close to 10.7 μm indicates the formation of consolidated macroflocs, which are more than 3–5 times larger than those observed during the intermediate aggregation stage, typically around 2–3 μm. This marked increase in particle size suggests the structural growth of electro-generated Fe­(OH)­x floc networks and supports the contribution of sweep coagulation as an important removal pathway.^52–54^

In Figure 8c, the zeta potential remained very close to zero, with average values of +1.9, + 1.4, and +0.9 mV, indicating that the suspension was already electrostatically destabilized after electrocoagulation. The narrow distribution further suggests surface-charge homogenization, probably associated with the incorporation of paracetamol and suspended species into ferric hydroxide flocs.^52,53^

Figure 8d presents hydrodynamic diameters of 2033, 1770.8, and 1958.9 nm, indicating the consolidation of a residual micrometric fraction within the 1.7–2.0 μm range. Unlike the more extreme macroflocculation behavior observed in Figure 8b, this distribution reflects a more uniform particle population, suggesting that after the sedimentation of larger macroflocs, a residual fraction of stable micrometric aggregates remains dispersed in the treated solution.^52–54^

Overall, after electrocoagulation, the system changed from an initially repulsive condition to a destabilized state with floc growth. The shift from approximately +20 mV toward 0 mV is consistent with charge neutralization.^52,53^ Diameters above 10 μm are consistent with sweep coagulation and physical entrapment in iron-hydroxide flocs, but they do not constitute direct molecular evidence for paracetamol incorporation.^54,55^

### 3.6 Comparative Techno-Economic Assessment under Conventional and Photovoltaic Power Supply

Table 8 presents a focused comparison of theoretical iron dissolution, reactor energy consumption, and partial variable cost at the maximum-removal condition (Run 20). The FCCCD optimization was conducted under the regulated conventional DC supply, whereas the selected condition was subsequently operated through the PV–MPPT–battery route. Accordingly, Table 8 is restricted to Run 20 and is not intended as a comparison across all FCCCD runs.

**Table 8:** Focused Comparison of Theoretical Iron Dissolution, Reactor Energy Consumption, and Partial Variable Cost for Run 20 Under Conventional and Photovoltaic Power Supply^a^

Indicator	Conventional	Photovoltaic
Condition	Run 20	Run 20
Theoretical iron dissolution (kg·m^–3^)	0.001737	0.001737
Reactor energy consumption (kWh·m^–3^)	0.018315	0.018315
Partial variable cost (BRL·m^–3^)	0.013482	0.000495

a Note: Values in Table 8 refer exclusively to Run 20. The photovoltaic partial variable cost (BRL 0.000495·m^–3^) represents a marginal-cost scenario in which a purchase price of zero was assumed for photovoltaic electricity. This value includes the cost associated with theoretical iron dissolution but excludes photovoltaic-system capital, the MPPT controller, battery replacement, maintenance, electrode cleaning or replacement, sludge management, labor, financing, and life-cycle costs. It should therefore not be interpreted as the total treatment cost.

For Run 20, the conventional and photovoltaic configurations were assigned the same physical reactor demand: 0.018315 kWh·m^–3^ and 0.001737 kg·m^–3^. This preserves the treatment condition and isolates the economic effect of the electricity-purchase-price assumption.

No arithmetic mean across operating scenarios was used. The photovoltaic result for Run 20 was calculated under an independent marginal-cost boundary in which the purchase price of photovoltaic electricity was set to zero, while reactor energy demand and theoretical iron dissolution remained unchanged. The comparison must therefore be interpreted only at the selected condition and within the stated cost boundary.

Run 20 achieved the highest experimental paracetamol removal (90.7%) and was therefore selected for the focused techno-economic comparison. Under this condition, the conventional and photovoltaic configurations showed the same reactor energy consumption (0.018315 kWh·m^–3^) and theoretical iron dissolution (0.001737 kg·m^–3^). When grid-purchased electricity was included, the conventional partial variable cost was BRL 0.013482·m^–3^. Under the marginal photovoltaic scenario, in which a purchase price of zero was assigned to solar electricity, the partial variable cost decreased to BRL 0.000495·m^–3^, corresponding to a reduction of 96.33%. This economic difference resulted exclusively from the assumed electricity purchase price.

The residual paracetamol concentration obtained in Run 20 was 18.6 mg·L^–1^. Therefore, this condition represents the most favorable removal–cost combination within the investigated experimental domain and the adopted marginal-cost boundary, but it does not demonstrate compliance with a discharge or water-quality criterion. No claim is made that the renewable origin of the electricity itself reduced reactor energy consumption, theoretical iron dissolution, or improved paracetamol removal.

Consequently, Table 8 provides a scenario-specific physical and economic assessment but does not constitute evidence that photovoltaic electricity intrinsically improves electrocoagulation performance. Photovoltaic power can replace electricity purchased from the grid and facilitate operation in remote locations; however, a comprehensive economic comparison should include the photovoltaic module, MPPT controller, battery replacement, maintenance, sludge management, labor, financing, and system lifetime. When selecting operating conditions, the electrical dose delivered to the reactor should first be minimized according to a predefined treatment objective. Subsequently, the photovoltaic system and energy-storage capacity should be sized using continuously recorded voltage and current profiles, operating time, and local solar irradiance. These estimates are useful for preliminary energy-demand analyses and system sizing, whereas pilot-scale validation should incorporate measured electrical profiles and a comprehensive life-cycle cost assessment.^56–59^

## 4 Conclusions

Within the investigated experimental domain, iron-electrode electrocoagulation achieved a maximum paracetamol removal of 90.7% at an initial concentration of 0.2 mg·mL^–1^, a reaction time of 30 min, a current density of 3.81 A·m^–2^, and an initial pH of 6.5. The corresponding residual concentration was 18.6 mg·L^–1^. Therefore, this condition represents the highest removal achieved within the experimental domain but does not, by itself, demonstrate compliance with a discharge or water-quality criterion.

The quadratic model was statistically significant (p = 0.0293; R^2^ = 0.7818), whereas the adjusted R^2^ of 0.5273 indicates moderate explanatory capacity. Therefore, the model is suitable for identifying interactions and favorable operating regions within the investigated ranges but not for extrapolation. The significant interactions between current density and pH, concentration and current density, concentration and pH, and reaction time and pH confirm that process efficiency depended on coupled electrical and chemical conditions.

Near-zero ζ-potential values and floc growth up to approximately 44 μm were consistent with colloidal destabilization, charge neutralization, and sweep coagulation. Nevertheless, these measurements constitute indirect physicochemical evidence and do not establish a unique molecular removal mechanism or confirm the complete mineralization of paracetamol.

The photovoltaic module, MPPT controller, and battery functioned exclusively as an alternative configuration for supplying electricity to the reactor and were not included as optimization factors. At the maximum-removal condition, the conventional and photovoltaic configurations exhibited the same reactor energy consumption of 0.018315 kWh·m^–3^ and the same theoretical iron dissolution of 0.001737 kg·m^–3^. However, within the marginal variable-cost scenario, photovoltaic energy was more cost-efficient than conventional electricity. When a marginal purchase price of zero was assigned to photovoltaic electricity, the partial variable cost decreased from BRL 0.013482·m^–3^ to BRL 0.000495·m^–3^, representing a reduction of 96.33%.

This economic advantage did not result from reduced energy consumption or theoretical iron dissolution but from the absence of a direct payment for photovoltaic electricity within the adopted cost boundary. The calculation excluded the capital costs of the photovoltaic module and MPPT controller, battery replacement, maintenance, electrode cleaning, sludge management, labor, financing, and life-cycle impacts. Consequently, this result should be interpreted as an advantage in marginal variable cost rather than as evidence of a lower total treatment cost.

The main limitations of the study include the use of a synthetic water matrix, the high initial and residual paracetamol concentrations, batch operation, the moderate predictive capacity of the model, uncontrolled photovoltaic current, and the absence of analyses of transformation products, mineralization, toxicity, sludge production, and life-cycle performance. Future studies should evaluate real wastewater and environmentally relevant concentrations, continuously record current and voltage, measure actual electrode mass loss, and conduct a comprehensive pilot-scale economic assessment.
